# Patient-derived and gene-edited pluripotent stem cells lacking *NPHP1* recapitulate juvenile nephronophthisis in abnormalities of primary cilia and renal cyst formation

**DOI:** 10.3389/fcell.2024.1370723

**Published:** 2024-06-26

**Authors:** Yutaka Arai, Hidenori Ito, Tomoya Shimizu, Yuzuno Shimoda, Dan Song, Mami Matsuo-Takasaki, Tadayoshi Hayata, Yohei Hayashi

**Affiliations:** ^1^ iPS Cell Advanced Characterization and Development Team, Bioresource Research Center, RIKEN, Tsukuba, Ibaraki, Japan; ^2^ Department of Molecular Pharmacology, Graduate School of Pharmaceutical Sciences and Faculty of Pharmaceutical Sciences, Tokyo University of Science, Chiba, Japan; ^3^ School of Integrative and Global Majors, University of Tsukuba, Tsukuba, Ibaraki, Japan

**Keywords:** nephronophthisis, iPS cells, induced pluripotent stem cells, NPHP1, kidney organoid, primary cilia, renal cyst

## Abstract

Juvenile nephronophthisis is an inherited renal ciliopathy with cystic kidney disease, renal fibrosis, and end-stage renal failure in children and young adults. Mutations in the *NPHP1* gene encoding nephrocystin-1 protein have been identified as the most frequently responsible gene and cause the formation of cysts in the renal medulla. The molecular pathogenesis of juvenile nephronophthisis remains elusive, and no effective medicines to prevent end-stage renal failure exist even today. No human cellular models have been available yet. Here, we report a first disease model of juvenile nephronophthisis using patient-derived and gene-edited human induced pluripotent stem cells (hiPSCs) and kidney organoids derived from these hiPSCs. We established NPHP1-overexpressing hiPSCs from patient-derived hiPSCs and NPHP1-deficient hiPSCs from healthy donor hiPSCs. Comparing these series of hiPSCs, we found abnormalities in primary cilia associated with *NPHP1* deficiency in hiPSCs. Kidney organoids generated from the hiPSCs lacking *NPHP1* formed renal cysts frequently in suspension culture with constant rotation. This cyst formation in patient-derived kidney organoids was rescued by overexpression of *NPHP1*. Transcriptome analysis on these kidney organoids revealed that loss of NPHP1 caused lower expression of genes related to primary cilia in epithelial cells and higher expression of genes related to the cell cycle. These findings suggested the relationship between abnormality in primary cilia induced by NPHP1 loss and abnormal proliferative characteristics in the formation of renal cysts. These findings demonstrated that hiPSC-based systematic disease modeling of juvenile nephronophthisis contributed to elucidating the molecular pathogenesis and developing new therapies.

## Introduction

Juvenile nephronophthisis (nephronophthisis type 1; NPH1) is an autosomal recessive kidney disease representing progressive renal dysfunction in childhood and young adults, leading to end-stage-renal-disease (ESRD) before 30s (Cantani et al., 1986). Several tens of genes have been identified as causal genes for nephronophthisis (Luo and Tao, 2018), and the varieties of gene mutations lead to the different time points of ESRD: infantile, juvenile, and adolescent (Petzold et al., 2023). The most frequent onset is in juveniles caused by NPHP1 gene mutation, accounting for 20%–25% of all cases (Halbritter et al., 2013; Braun and Hildebrandt, 2017; Leggatt et al., 2023). The critical symptom in juvenile nephronophthisis is the formation of many cysts in the kidney medullar, causing functional disorder of nephrons depending on the collapse of kidney morphology (Raina et al., 2023). Some patients occasionally exhibit extra-renal manifestations including dysfunction of eyes, central nervous system, liver, bone, heart, and lung (Otto et al., 2003; Gunay-Aygun, 2009; Baujat et al., 2013; Barker et al., 2014; Kang et al., 2015). Although symptomatic treatment, including dialysis, is applied to nephronophthisis, no curative therapies exist except for kidney transplantation (Devlin et al., 2023).

The causal gene *NPHP1* encoding nephrocystin-1 is related to primary cilium formation. Thus, NPH1 is defined as a form of ciliopathy (Novarino et al., 2011). Nephrocystin-1, located at the transition zone of primary cilia, functions as a regulator of signal transduction and protein transport between cilium and cytoplasm by forming complexes with other cilium-related proteins (Garcia-Gonzalo et al., 2011; Sang et al., 2011; Chaki et al., 2012; Gupta et al., 2015; Shi et al., 2017). In a previous study, *Nphp1*-knockout (KO) mice gradually exhibited and expanded renal cysts at 5 months after birth (Li et al., 2021; Garcia et al., 2022); however, little is known yet about the molecular mechanisms of how nephrocystin-1 deficiency causes cyst formation in kidneys and leads to ESRD. Therefore, NPH1 models based on human cells should be required to reveal the molecular pathogenesis and to develop new therapeutic approaches to prevent the formation and expansion of renal cysts.

To investigate the pathomechanisms of juvenile nephronophthisis in humans, we have focused on utilizing human induced pluripotent stem cells (hiPSCs). hiPSCs generated from somatic cells of patients suffered from intractable diseases are expected to be valuable bioresources to recapitulate these symptoms at the cellular level. In our previous study, we generated and characterized two juvenile nephronophthisis-specific hiPSC lines derived from two patients carrying deletions spanning the whole NPHP1 gene (Arai et al., 2020). Recently, other researchers reported the generation of NPHP1-KO hiPSCs (Nakano et al., 2022; Dyke et al., 2023). However, no human disease models of juvenile nephronophthisis have been established to date. Human disease modeling of polycystic kidney disease (PKD), which exhibits similar cystic phenotypes to nephronophthisis has been successfully demonstrated using hiPSCs-derived kidney organoids (Low et al., 2019; Kuraoka et al., 2020; Li et al., 2022; Tran et al., 2022). Also, infant nephronophthisis has been recapitulated with kidney organoids from only one patient-derived hiPSC line (Forbes et al., 2018). Thus, we have hypothesized that disease modeling for juvenile nephronophthisis can be established using kidney organoids generated from patient-derived and gene-edited hiPSCs carrying NPHP1 deficiency.

In this study, we have generated NPHP1-overexpressing (OE) hiPSCs from patient-derived hiPSCs by the PiggyBac transposon system. In addition, we developed homozygous NPHP1-deficient hiPSCs from healthy donor hiPSCs using CRISPR-Cas9 gene editing. Using these hiPSCs, we examined the morphology of primary cilia and found clear effects of NPHP1 loss. We also generated kidney organoids from these hiPSCs and examined their morphology and transcriptome. The formation of renal cysts in these kidney organoids carrying NPHP1 loss was successfully recapitulated and supported in the differential gene expression patterns in the transcriptome analysis. Our systematic disease modeling of juvenile nephronophthisis contributes to elucidating the molecular pathogenesis and developing new therapies.

## Results

### Generation of NPHP1-overexpressing hiPSCs derived from patients’ hiPSCs with whole NPHP1 deletion

**FIGURE 1:**
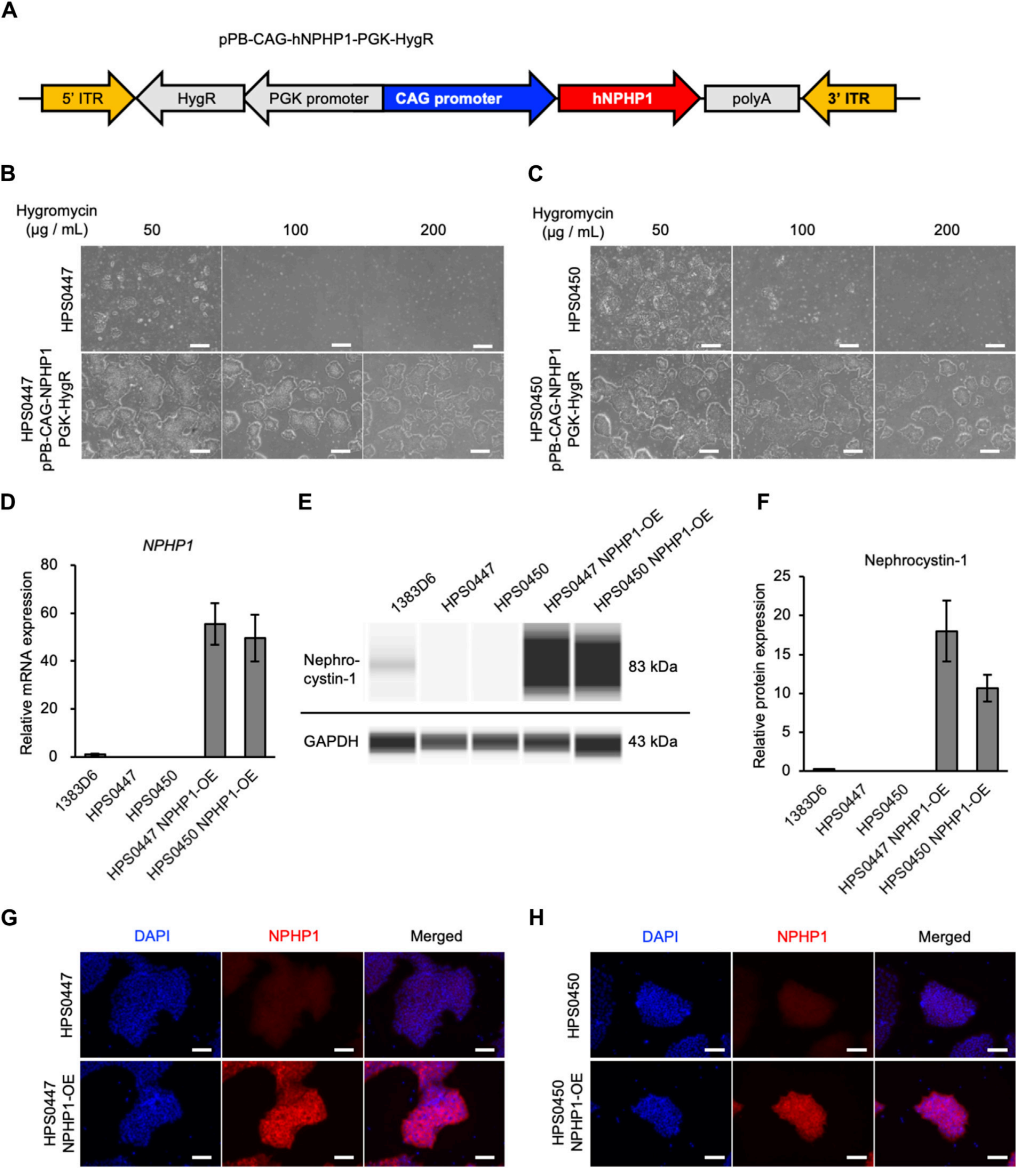
Generation of NPHP1-overexpressing hiPSCs derived from patient-derived hiPSCs with whole NPHP1 deletion. **(A)** Schematic construct of pPB-CAG-NPHP1-PGK-HygR in DNA transposon. pPB: piggybac transposon plasmid, CAG: Cytomegalovirus (CMV) early enhancer element, the promoter, the first exon and the first intron of chicken beta-Actin gene, and the splice acceptor of the rabbit beta-Globin gene, PGK: phosphoglycerate kinase, HygR: hygromycin resistant gene. **(B,C)** Phase contrast images of patient-derived hiPSCs and their transfected cells treated with hygromycin at 50, 100, and 200 μg/mL for 5 days. Scale bars = 500 µm. **(D)** Relative mRNA expression of *NPHP1* in healthy donor hiPSCs (1383D6 line), patient-derived hiPSC lines, HPS0447 and HPS0450, transfected hiPSC lines. HPS0447 NPHP1-OE and HPS0450 NPHP1-OE. Each value was normalized with GAPDH value from the same sample. Data are shown as the mean ± SE (n = 3). **(E)** Representative images of an automatic capillary Western blot on nephrocystin-1 protein in healthy donor hiPSCs (1383D6 line), patient-derived hiPSC lines, HPS0447 and HPS0450, transfected hiPSC lines, HPS0447 NPHP1-OE and HPS0450 NPHP1-OE. **(F)** Relative protein expression of nephrocystin-1 calculated from the data of automatic capillary Western blot. Each value was normalized with GAPDH value from the same sample. Data are shown as the mean ± SE (n = 3). **(G,H)** Immunocytochemistry of NPHP1 in patient-derived hiPSC line, HPS0447 and HPS0450, transfected hiPSC lines, HPS0447 NPHP1-OE and HPS0450 NPHP1-OE. Nuclei are stained with DAPI. Scale bars = 100 µm.

In our previous study (Arai et al., 2020), we generated and characterized hiPSC lines derived from two NPH1 patients to enable disease modeling. To validate the functional effects of loss-of-NPHP1 in these patient cells and establish the rescued models, we introduced the *NPHP1* gene in these patient-derived hiPSCs with whole *NPHP1* gene deletion. We first constructed a PiggyBac transposon vector expressing the *NPHP1* gene driven by CAG promoter and hygromycin resistance gene driven by PGK promoter. This vector and PiggyBac transposase vector were transfected to patient-derived hiPSC lines (Tanaka et al., 2013) (Figure 1A). Transfected hiPSCs were treated with hygromycin and selected in a dose-dependent manner (Figures 1B, C). Only transfected hiPSCs survived with 200 μg/mL of hygromycin. We then examined whether the *NPHP1* gene and its coding protein nephrocystin-1 were expressed in these transfected and selected hiPSCs derived from patient-derived hiPSCs. Although the expression of the *NPHP1* gene was absent in patient-derived hiPSCs, transfected hiPSCs expressed it at considerable levels (Figure 1D). Nephrocystin-1 protein was certainly missing in both patient-derived hiPSCs; however, the expression was detected in both transfected hiPSCs (Figures 1E, F). Furthermore, the expression of nephrocystin-1 was observed in both transfected hiPSCs detected with immunocytochemistry (Figures 1G, H). These results indicated that NPHP1-OE hiPSCs derived from patient-derived hiPSCs were successfully generated by the PiggyBac transposon system and rescued loss of *NPHP1* in the expression at mRNA and protein levels.

### Generation of NPHP1-deficient hiPSCs derived from healthy donor hiPSCs

**FIGURE 2:**
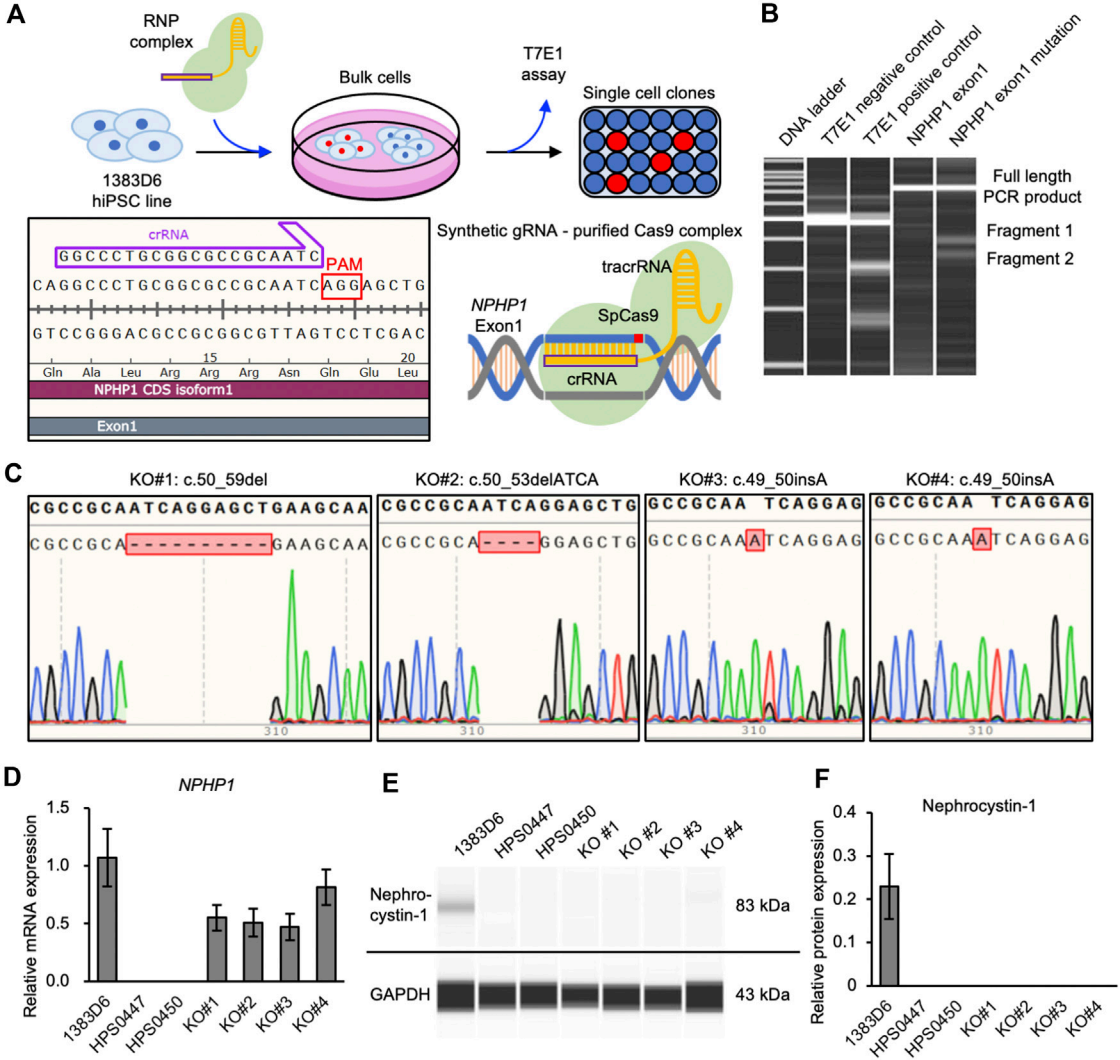
Generation of NPHP1-deficient hiPSCs derived from healthy donor hiPSCs. **(A)** Schematics of the generation of NPHP1-deficient hiPSCs by CRISPR-Cas9 system using synthetic RNA-protein complex. **(B)** Microchip-based electrophoresis of DNA bands of PCR products of NPHP1 exon1 cleaved in T7E1 assay. **(C)** Genomic sequences of exon one of *NPHP1* with insertion or deletion in genome-edited hiPSCs. **(D)** Relative mRNA expression of *NPHP1* in healthy donor hiPSCs (1383D6 line), patient-derived hiPSCs HPS0447 and HPS0450 lines, gene-edited hiPSC clones, KO#1, KO#2, KO#3, and KO#4. Each value was normalized with GAPDH value from the same sample. Data are shown as the mean ± SE (n = 3). **(E)** Representative images of Western blot on nephrocystin-1 protein in healthy donor hiPSCs (1383D6 line), patient-derived hiPSCs HPS0447 and HPS0450 lines, gene-edited hiPSC clones, KO#1, KO#2, KO#3, and KO#4. **(F)** Relative protein expression of nephrocystin-1 normalized with expression of GAPDH in healthy donor hiPSCs 1383D6 (n = 3), patients’ hiPSCs HPS0447 (n = 3) and HPS0450 (n = 3), genome-edited hiPSCs KO#1, KO#2, KO#3, KO#4 (n = 3, respectively). Data are shown as the mean ± SE (n = 3).

To more rigorously evaluate the effects of loss of *NPHP1* between isogenic pairs of hiPSC lines, we generated NPHP1-deficient hiPSCs derived from healthy donor hiPSCs using the CRISPR-Cas9 system (Xu et al., 2021) (Figure 2A). We designed guide RNA targeting exon one of the *NPHP1* gene without any potential complete 20-nt off-target sequences in the human genome. Ribonucleoprotein (RNP) complex of the guide RNA and S.p. Cas9 protein were transduced to induce homozygous DNA cleavage and non-homologous end joining causing insertion or deletion (indel) mutations in *NPHP1* gene, to healthy donor hiPSCs. After transfection, the efficiency of genome editing was evaluated with the T7E1 assay, which detects unmatched DNA strands caused by gene-edited mutations. We detected cleaved PCR products of exon one in the *NPHP1* gene, suggesting that the *NPHP1* gene was efficiently edited in the transfected cell population (Figure 2B). Then, we expanded single cell-derived clones and examined the genomic sequence of exon one region in NPHP1 gene. Among 48 clones examined, 20 and four hiPSC clones carried heterozygous and homozygous mutations at the protein-coding sequence of exon one in the *NPHP1* gene, respectively (Figure 2C). The overall genome editing efficiency was 29.2% at allele basis. We examined the expression of *NPHP1* mRNA and nephrocystin-1 protein in the homozygous hiPSC clones with quantitative RT-PCR and automatic capillary Western blotting, respectively. The expression of mRNA in these homozygous hiPSC clones was reduced compared to the original hiPSC line possibly due to RNA decay (Figure 2D). The protein expression of nephrocystin-1 was lost in gene-edited hiPSCs (Figures 2E, F). These results suggested that frameshift mutation of the exon one in the *NPHP1* gene induced by CRISPR-Cas9 genome editing successfully led to the generation of isogenic NPHP1-deficient hiPSC lines derived from a healthy donor hiPSC line.

### Loss of NPHP1 abnormally prolongs the cilia and decreases the ratio of ciliated cells in hiPSCs

**FIGURE 3:**
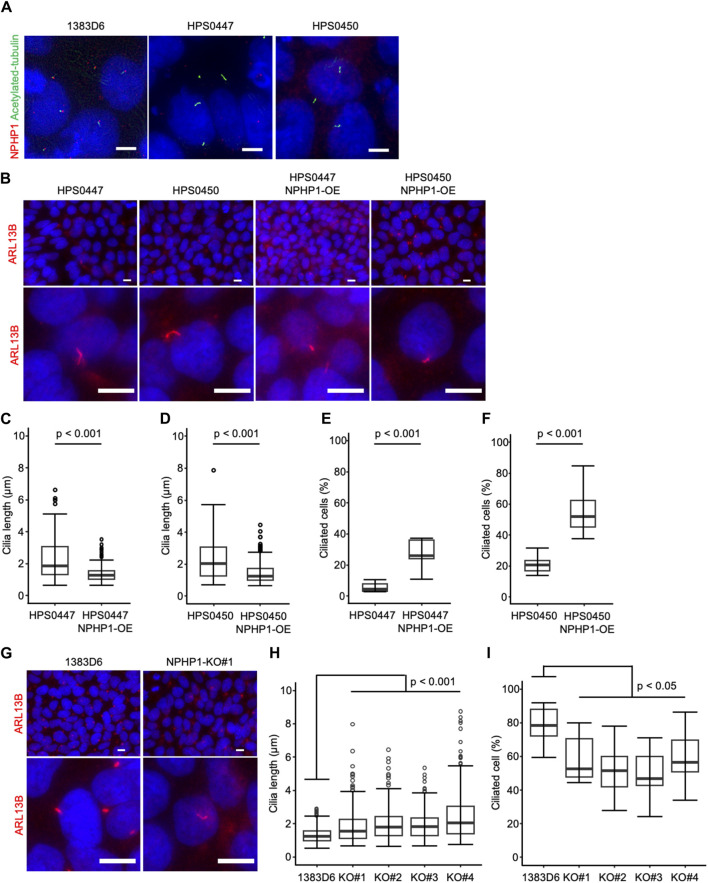
Loss of NPHP1 abnormally prolongs the cilia and decreases the ratio of ciliated cells in human iPS cells. **(A)** Immunocytochemistry of NPHP1 and acetylated-tubulin proteins in healthy donor hiPSCs (1383D6 lines), patient-derived hiPSC lines, HPS0447 and HPS0450. Nuclei are stained with DAPI. Scale bars = 5 µm. **(B)** Immunocytochemistry of ARL13B protein in healthy donor hiPSCs (1383D6 line), patient-derived hiPSC lines, HPS0447 and HPS0450, NPHP1-overexpressing hiPSC lines, HPS0447 NPHP1-OE and HPS0450 NPHP1-OE, NPHP1-deficient hiPSC lines NPHP1-KO. Lower panels exhibit the magnified images. Scale bars = 10 µm. **(C,D)** Length of primary cilia in patients’ hiPSCs HPS0447 (n = 72) and HPS0450 (n = 70), NPHP1-overexpressing hiPSCs HPS0447 NPHP1-OE (n = 111) and HPS0450 NPHP1-OE (n = 188) shown with a box-and-whisker plot. *p*-values were determined by an unpaired two-tailed Student’s t-test. **(E,F)** The ratio of ciliated cells in patients’ hiPSCs HPS0447 and HPS0450, NPHP1-overexpressing hiPSCs HPS0447 NPHP1-OE and HPS0450 NPHP1-OE. The ratio of ciliated cells was calculated from each image normalized by DAPI and shown using a box-and-whisker plot (n = 10). *p*-values were determined by an unpaired two-tailed Student’s t-test. **(G)** Immunocytochemistry of ARL13B protein in healthy donor hiPSCs (1383D6 line), NPHP1-deficient hiPSC lines NPHP1-KO. Lower panels exhibit the magnified images. Scale bars = 10 µm. **(H)** Length of primary cilia in healthy donor hiPSCs (1383D6) (n = 272), NPHP1-deficient hiPSC clones KO#1 (n = 246), KO#2 (n = 195), KO#3 (n = 205), and KO#4 (n = 250) shown using a box-and-whisker plot. *p*-values were determined by Tukey’s test. **(I)** Ratio of ciliated cells in healthy donor hiPSCs 1383D6 (n = 10), NPHP1-deficient hiPSCs KO#1 (n = 10), KO#2 (n = 10), KO#3 (n = 10), KO#4 (n = 10). The ratio was calculated from individual images (n = 10) normalized by DAPI and shown using a box-and-whisker plot. *p*-values were determined by Tukey’s test.

To examine the effect of loss of *NPHP1* in hiPSCs, we first focused on primary cilia. Previous studies showed that renal epithelial cells from juvenile nephronophthisis patients carried abnormal cilia, likely to cause renal dysfunction (Ning et al., 2021; Garcia et al., 2022). Therefore, we asked whether NPHP1-deficient hiPSCs showed cilia abnormality. Since the formation and morphology of cilia were affected by the cell cycle (Tucker et al., 1979; Pan and Snell, 2007; Saito et al., 2017; Sakaji et al., 2023), we examined the cell proliferation rate as the cell number on day 7 after each passage of undifferentiated hiPSCs. There were no detectable differences in cell proliferation rate between patients-derived hiPSCs and NPHP1-OE hiPSCs and between healthy control hiPSCs and NPHP1-deficient hiPSC clones (Supplementary Figures S1A–C). These results indicated there were no effects of *NPHP1* expression on the proliferation or cell cycle rate in undifferentiated hiPSCs. We then examined the expression of nephrocystin-1 and a marker protein for primary cilia, acetylated-tubulin, in hiPSCs detected with immunocytochemistry. Although the foci of nephrocystin-1 expression were situated near the basal position of primary cilia in healthy donor hiPSCs, these patient-derived hiPSCs had no or little foci of nephrocystin-1 near cilia (Figure 3A). We further measured the cilium length with cellular images from immunocytochemistry using antibody against a cilia-specific protein, ARL13B (Figure 3B). The overexpression of NPHP1 in patient-derived hiPSC lines significantly shortened the length of cilia (Figures 3C, D) and significantly increased the ratio of ciliated cells (Figures 3E, F). To more rigorously re-evaluate the effects of expression change of NPHP1 on cilium morphology, we next asked whether the deficiency of NPHP1 prolonged the length of cilia and reduced the ratio of ciliated cells among isogenic iPSC lines. The loss of NPHP1 significantly extended the length of cilia and reduced the ratio of ciliated cells in hiPSCs (Figures 3G–I). These results suggested that the deficiency of NPHP1 caused abnormal morphology of cilia in undifferentiated hiPSCs and that the overexpression of NPHP1 in patients could partially rescue the abnormality of cilia conversely.

### Kidney organoids derived NPHP1-deficient hiPSCs recapitulate the renal cyst formation

**FIGURE 4:**
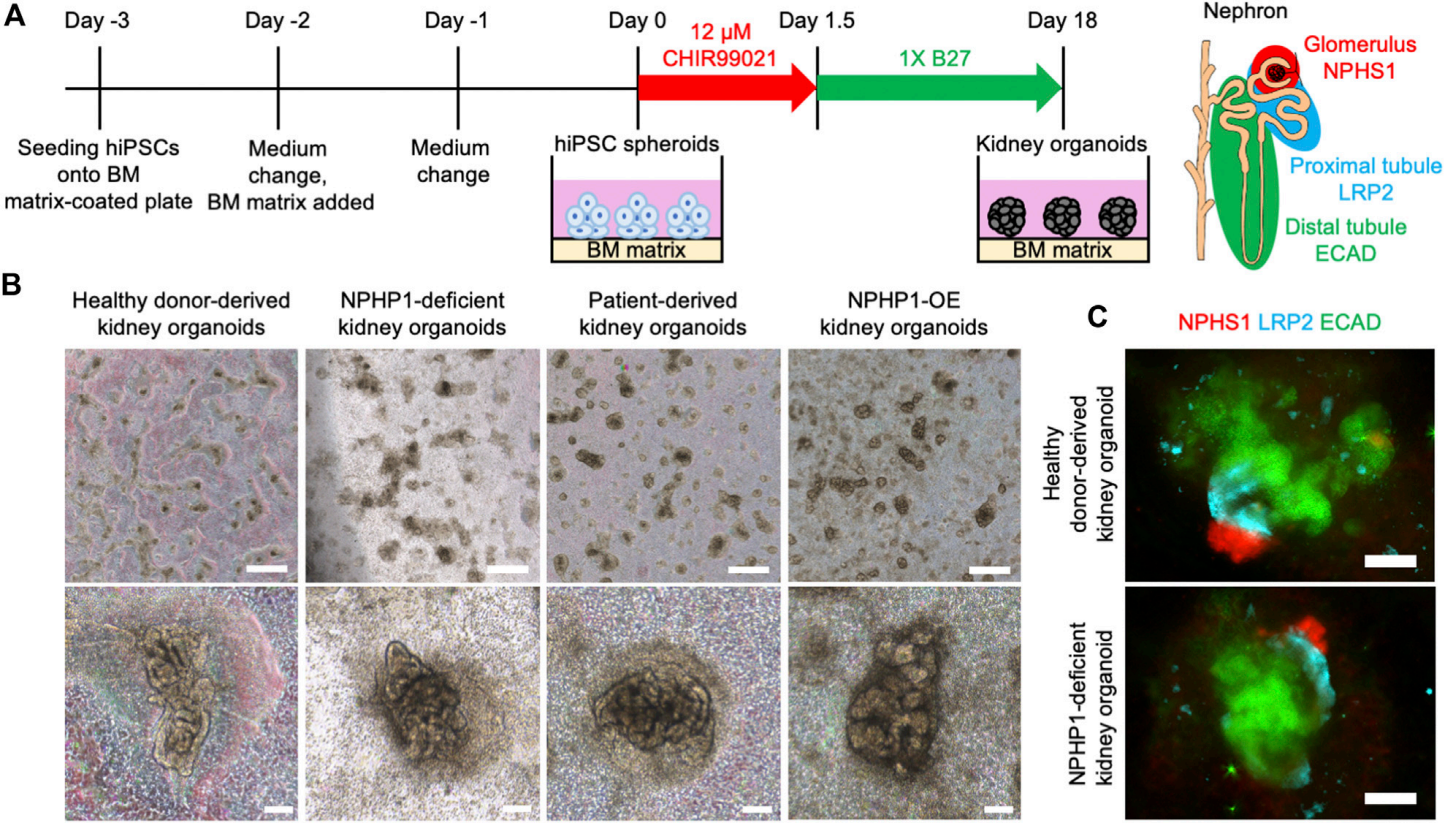
Kidney organoids derived NPHP1-deficient hiPSCs formed normally in the initial phase. **(A)** Schematics of the generation of kidney organoids from hiPSCs. **(B)** Phase contrast images of kidney organoids on day 18 from iPSC lines of 1383D6, NPHP1-KO#2, HPS0450, and HPS0450 NPHP1-OE. Scale bars = 1 mm (upper panels) and 100 µm (bottom panels). **(C)** Immunocytochemistry of nephron markers in kidney organoids derived from healthy donor hiPSCs (1383D6 line) and NPHP1-deficient hiPSCs (NPHP1-KO#2) on day 18. NPHS1^+^ cells, LRP2^+^ cells, and ECAD^+^ cells suggest glomeruli, proximal tubule, and distal tubule, respectively. Scale bars = 100 µm.

Renal cyst formation is a major symptom of juvenile nephronophthisis, which disrupts nephron tissues and causes end-stage renal disease (Luo and Tao, 2018; Petzold et al., 2023). Thus, we aimed to recapitulate the renal cyst formation using kidney organoids derived from hiPSCs. We generated kidney organoids from the hiPSCs referring to previous reports with minor modification (Freedman et al., 2015; Cruz et al., 2017) (Figure 4A). On the differentiation day 18, nephron structures including glomeruli marked by NPHS1 expression and proximal and distal tubules, marked by the expression of LRP2 and ECAD respectively, were formed from healthy donor hiPSCs, NPHP1-deficient hiPSCs, patient hiPSCs and NPHP1-OE hiPSCs (Figures 4B, C).

**FIGURE 5:**
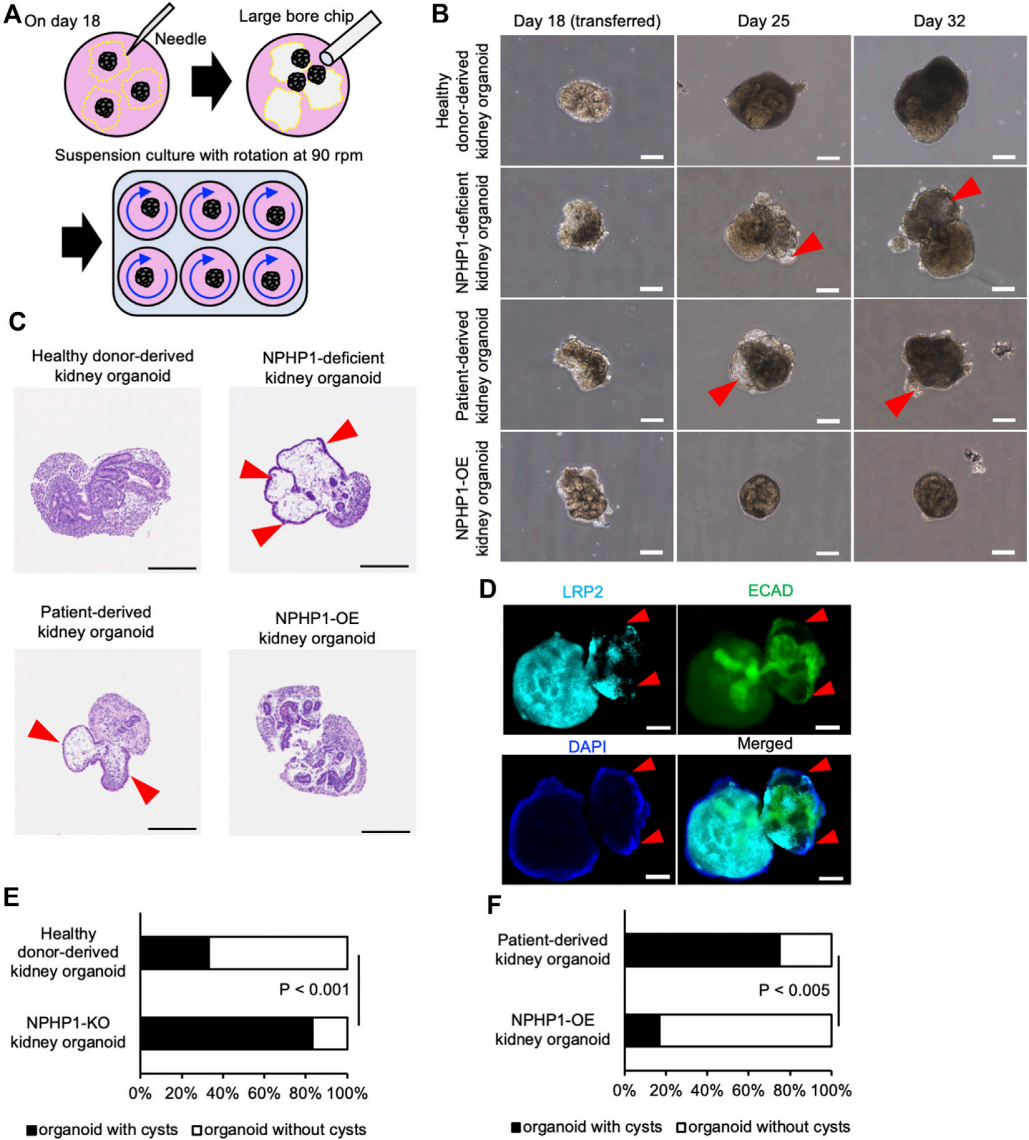
Kidney organoids derived NPHP1-deficient hiPSCs recapitulate the renal cyst formation in suspension culture with rotation. **(A)** Schematics of suspension culture of kidney organoids. **(B)** Representative phase contrast images of kidney organoids from the same iPSC lines in Figure 4B in suspension culture on days 18, 25, and 32. Red arrowheads indicate renal cysts. Scale bars = 200 µm. **(C)** Representative bright field images of HE-stained sections of kidney organoids derived from iPSC lines of 1383D6, NPHP1-KO#2, HPS0450, and HPS0450 NPHP1-OE lines on day 25. Red arrowheads indicate renal cysts. Scale bars = 200 µm. **(D)** Immunocytochemistry of tubule markers in a cystic kidney organoid on day 25 from NPHP1-KO#2 line. Nuclei are stained with DAPI. Red arrowheads indicate expansion sites of tubule structure. The white arrowhead indicates cell aggregation formed by suspension culture. Scale bars = 100 µm. **(E,F)** Comparison of the ratio of cyst formation in kidney organoids by day 32 between healthy donor hiPSCs (1383D6 line) and NPHP1-KO#4 clone (E; n = 24) and between patient-derived iPSC line, HPS0450, and HPS0450 NPHP1-OE (F; n = 12). The cysts were identified as a structure protruding from the nephron tissue and surrounded by epithelial-like cells, which could be clearly observed by phase contrast microscopy images. *p*-values were determined by Pearson’s chi-square test.

To determine whether these kidney organoids formed renal cysts or not, they were then exposed to continuous fluid stress in a suspension culture with rotation to mimic the constant flow of raw urine in nephrons. On the differentiation day 18, kidney organoids were manually picked up from the adherent culture and transferred to the suspension culture dishes with continuous rotation at 90 rpm (Figure 5A). After 1 week in the suspension culture (on day 25), cyst formation was observed in NPH1 patient-derived kidney organoids and NPHP1-deficient kidney organoids (Figure 5B). Hematoxylin and eosin (HE) staining on the sections of these organoids revealed that these cysts were surrounded by epithelial cells (Figure 5C). Immunocytochemistry of LRP2 and ECAD indicated that these epithelial cells may originated from ECAD-positive distal tubule-like cells (Figure 5D). After 2 weeks in suspension culture (on day 32), around 80% of the patient-derived kidney organoids and NPHP1-deficient kidney organoids formed cysts although only 20%–30% of the isogenic kidney organoids and NPHP1-OE kidney organoids formed cysts (Figures 5E, F; Supplementary Figure S2). These results suggested that kidney organoids lacking NPHP1 exhibited renal cyst formation induced by continuous fluid stress and that overexpression of NPHP1 in patient-derived kidney organoids rescued the cyst formation.

### Transcriptome analysis of kidney organoids reveals that loss of NPHP1 causes primary cilia abnormalities in epithelial cells

**FIGURE 6:**
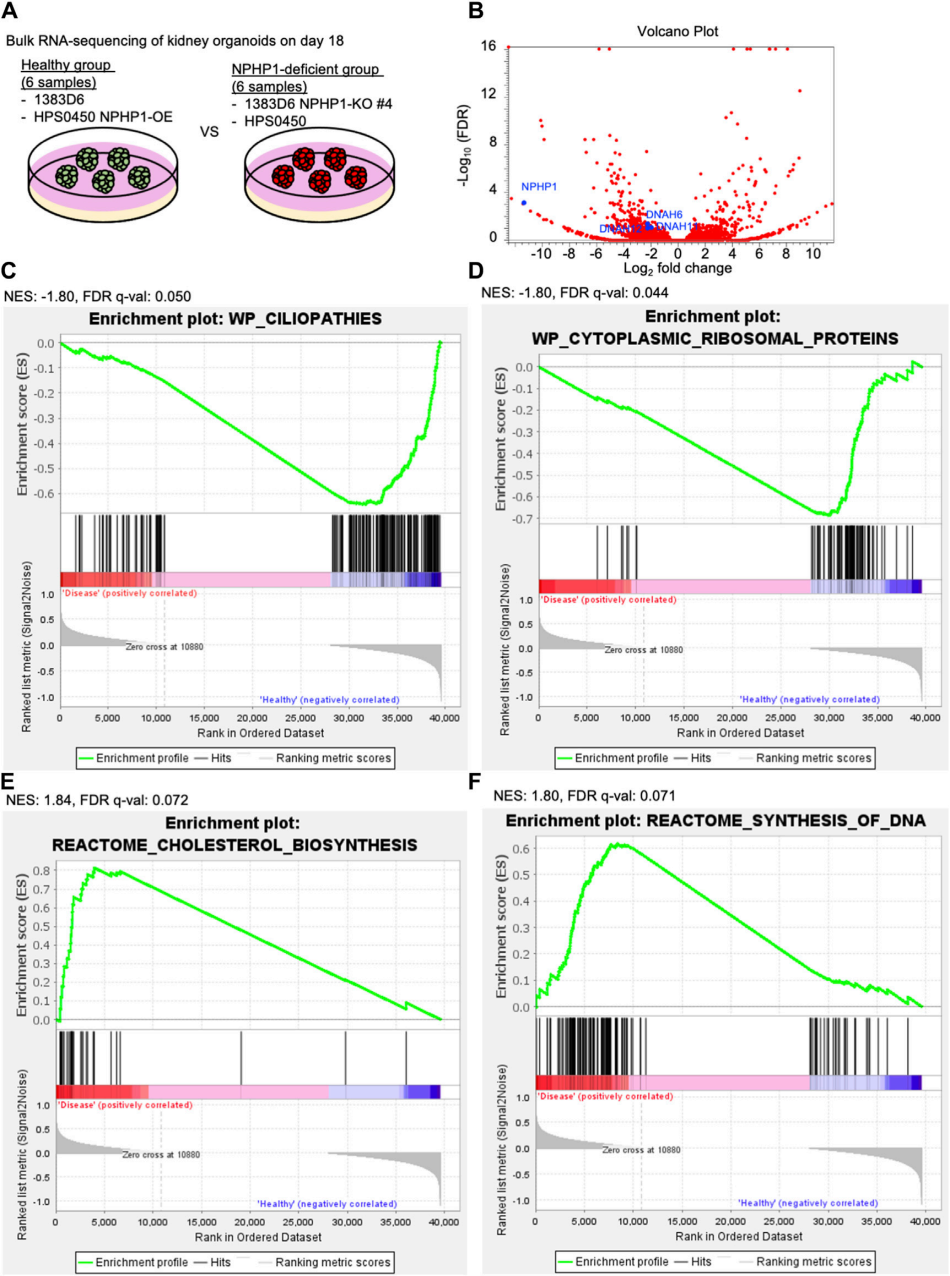
Transcriptome analysis of kidney organoids reveals ciliopathy features. **(A)** Schematics of bulk RNA-seq transcriptome on kidney organoids on day 18. 1363D6 (n = 3) and HPS0450 NPHP1-OE (n = 3) are divided into the healthy group and 1383D6 NPHP1-KO #4 (n = 3) and HPS0450 (n = 3) are divided into the disease group. **(B)** Volcano plot showing differential expression analysis compared to the healthy versus disease group. **(C–F)** Gene Set Enrichment Analysis (GSEA) showing enrichment scores (ES) of genes related to ciliopathies. The *X*-axis shows the individual genes (vertical black lines) in a gene set of ciliopathies. The *Y*-axis represents the enrichment score. The colored band at the bottom represents the degree of correlation of genes with the ciliopathies (red for positive and blue for negative correlation). The enrichment profile (green line) representing the negative curve of ES indicates an inverse correlation in the disease group. NES: normalized enrich score.

To explore the molecular mechanisms of cyst formation in kidney organoids lacking NPHP1, we performed RNA-seq transcriptome experiments. Because the potential for cyst formation by suspension culture should already be derived from kidney organoids at the adherent culture, we compared the global gene expression pattern of kidney organoids on day 18 between healthy group and NPHP1-deficient group (Figure 6A). The differential expression analysis identified that the expression of 211 genes including cilia-related genes *DNAH6*, *DNAH11*, *DNAH12* which code axonemal dynein known as major proteins composing cilia (Roberts et al., 2013; Sung and Leroux, 2013) as well as *NPHP1*, were significantly downregulated in the disease group (Figure 6B). A gene set enrichment analysis (GSEA) performed on the entire set of transcriptome data identified that the genes related to ciliopathies were negatively enriched in the NPHP1-deficient group (Figure 6C). These results indicated that kidney organoids lacking *NPHP1* reflected their disease phenotypes in their global gene expression patterns in an unbiased manner. Interestingly, the GSEA also identified that the genes related to cytoplasmic ribosomal proteins were negatively enriched in the NPHP1-deficient group and that cholesterol biosynthesis and cell cycle-related genes were positively enriched in the NPHP1-deficient group (Figures 6C–F; Supplementary Table S1). These results suggested that these signaling pathways and biological processes might be related to renal cyst formation.

We then performed gene ontology analysis (GOA; Metascape Gene List Analysis) for these significantly downregulated genes. As expected, GOA identified that these downregulated genes were related to cilium movement, especially in epithelial cilium movement involved in the extracellular fluid movement (Supplementary Figure S3A). According to GOA in Cell Type Signatures, these genes were enriched in fetal ciliated epithelial cells (Supplementary Figure S3B). GOA in DisGeNET emphasized that these genes were related to inherited ciliopathies in the whole body, such as Kartagener Syndrome, Microphthalmos and Primary Ciliary Dyskinesia (Supplementary Figure S3C). These results suggested that kidney organoids lacking *NPHP1* dysregulated genes related to abnormalities of cilia in epithelial cells. Together, transcriptome analysis supported that kidney organoids lacking *NPHP1* recapitulated the molecular pathogenesis of NPH1.

## Discussion

In this study, we have established a systematic disease model of juvenile nephronophthisis using human cultured cells for the first time. Although kidney organoids lacking *NPHP1* were successfully generated from hiPSCs without any apparent abnormalities in their structure at the initial period of adherent culture, cyst formation in these kidney organoids was induced by continuous fluid stress in suspension culture. These processes might reflect the initial pathological processes that lead to the juvenile onset due to the stress of the constant flow of primary urine in the nephrons of NPH1 patients. Likewise, previous studies showed that flow in suspension culture enhanced cyst formation from kidney organoids derived from PKD patients (Cruz et al., 2017; Li et al., 2022). Kidney organoids generated from *NPHP1*-deficient hiPSCs markedly formed cysts. Furthermore, the overexpression of *NPHP1* rescued the renal cyst formation in these patient-derived hiPSC lines. These results suggest that renal cyst formation in juvenile nephronophthisis could be prevented by the exogenous expression of NPHP1 as the proof-of-concept of gene therapy for NPH1. Although current techniques for kidney organoids are limited in their long-term monitoring because of the gradual necrosis and destruction of nephron structures in culture. Nutrient supply through appropriate vascularization and perfusion culture system may ameliorate this issue to observe further disease progression *in vitro*.

As for the molecular pathogenesis of NPH1 revealed from kidney organoids, our transcriptome analysis identified possible key features. GSEA and GOA analyses validate the dysregulation of genes related to ciliopathies and epithelial cilium movement in NPHP1-deficient kidney organoids. It might be interesting to investigate how the loss of *NPHP1* in renal epithelial cells leads to the abnormal expression patterns of other cilia-related genes at mRNA levels. We also identified that the expression of cell cycle-related genes was upregulated in NPHP1-deficient kidney organoids. This feature might reflect that epithelial cell proliferation was enhanced during renal cyst formation in NPH1 patients and NPHP1-deficient mouse models (Cantani et al., 1986; Luo and Tao, 2018; Li et al., 2021; Garcia et al., 2022; Petzold et al., 2023). Since we did not observe any differences in cell growth rate in an undifferentiated state in NPHP1-deficient hiPSCs, the cell cycle changes induced by NPHP1 might be cell-type specific. Furthermore, we identified that the genes related to cytoplasmic ribosomal proteins were negatively enriched in the NPHP1-deficient group and that cholesterol biosynthesis was positively enriched in the NPHP1-deficient group. NPHP1 could take a part in mechanosensory functions in the transition zone of the primary cilium to sense the fluid shear stress of urine flow and to transmit its signal through its interacting proteins to cope with protein translation and cholesterol biosynthesis (Garfa Traoré et al., 2023). NPHP1 deficiency might cause the abnormality in these pathways to lead to renal cyst formation as the molecular pathogenesis of NPH1. Also, it will be interesting to examine the role of other NPHP proteins that interact with NPHP1 protein using hiPSC-derived models with genome editing technologies for understanding the genetic heterogeneity of the nephronophthisis.

We found that loss of NPHP1 caused structural abnormalities of primary cilia in hiPSCs. In previous studies, primary epithelial cells and tissues in juvenile nephronophthisis patients and mice disease models lacking *NPHP1* have abnormally prolonged cilia and little number of ciliated cells (Ning et al., 2021; Garcia et al., 2022). We also demonstrated that the overexpression of *NPHP1* in the NPH1 patient-derived iPSCs partially rescued the cilia length and the ratio of ciliated cells (Figures 3C–F); however the ratio in the OE conditions did not reach the level of the healthy control iPSCs, 1383D6 line (Figures 3H, I). In the patient lines, homozygous deletions were identified involving the entire *NPHP1* gene and part of the next *MALL* gene region, and other unidentified factors during reprogramming into iPSCs might affect the formation of primary cilia. Taking into account the inter-donor and inter-line differences in cilia length, overexpression and knockout experiments to compare with the original line should be useful to determine the effect of NPHP1 gene. It is interesting to investigate how the structural abnormalities of primary cilia lead to renal cyst formation using hiPSC-derived kidney models.

In conclusion, our disease model provides the platform to model and elucidate a human micro-pathomechanism of juvenile nephronophthisis and to develop novel therapies. Conventional animal models such as *Nphp1*-KO mice hold the advantage of examining systemic symptoms and their impact on lifespan (Li et al., 2021; Garcia et al., 2022); however, it takes a long time to develop symptoms using these model mice. Previous immortal cell models are easy to manipulate and to examine the molecular mechanisms related to NPHP1 (Garcia-Gonzalo et al., 2011; Sang et al., 2011; Chaki et al., 2012; Gupta et al., 2015; Shi et al., 2017); however, it is hard to recapitulate the complex tissue organization and degeneration, such as nephrogenesis and cyst formation in kidneys. Kidney organoids derived from patient-specific hiPSCs should be beneficial in the development of patient-specific treatments and personalized medical strategies. Our novel disease model revealed that hiPSCs lacking NPHP1 recapitulated the phenotypes, suggesting that the abnormalities of cilia would convince the possibility for disorders of the whole body because of the character of iPSCs for pluripotency into three germ layers. In particular, since retinal disorders are known complications with juvenile nephronophthisis, our disease modeling system using NPHP1-hiPSCs can apply to many other ciliopathies such as eye disorders. We believe that our disease model will be useful in developing a treatment for NPH1, either through gene therapy by supplementing NPHP1 or through drug screening to prevent or stop cyst formation.

## Materials and methods

### Human subjects

The generation and use of hiPSCs in this study were approved by the Ethics Committees of RIKEN BioResource Research Center. Formal informed consent was obtained from the patients.

### Plasmid construction

The PiggyBac transposon vector system was used to integrate the human *NPHP1* gene into the genome DNA of patients’ hiPSC lines lacking the whole *NPHP1* gene. The PiggyBac plasmid vector pPB-CAG-NPHP1-PGK-Hyg^R^ used in this study was generated by In-Fusion cloning. To clone the human *NPHP1* CDS (CoDing Sequence), total RNA was extracted from hiPSC line 1383D6 with FastGene RNA premium kit (Nippon Genetics). cDNA was synthesized by RT-PCR using ReverTra Ace (TOYOBO). For In-Fusion cloning, two primer sets with 15 bp overlapping regions were designed for PCR, producing a cDNA fragment of *NPHP1* and linearized vector of pPB-PGK-destination (#60436, Addgene). The sequences of primers for the cDNA fragment and linearized vector are listed in Supplementary Table S2, respectively. The PCR was performed using PrimeSTAR Max DNA Polymerase (TAKARA) with the thermal cycling conditions as follows: initial denaturation at 94°C for 1 min, 35 cycles of 3-step thermo-cycling (denaturation at 98°C for 10 s, annealing at 60°C for 5 s and extension at 72°C for 5 s/kbp), and then hold at 4°C. PCR products were confirmed by MultiNA (SHIMAZU) and purified with FastGene Gel/PCR extraction kit (Nippon Gene). Purified two types of PCR products were annealed with In-Fusion enzyme (TAKARA) at 50°C for 15 min, which were further transformed into DH5a competent cells (SMOBIO TECHNOLOGY). The constructed plasmid vector was sequenced by Sanger sequencing at FASMAC. The primers to check the sequence of vector are listed in Supplementary Table S2. The sequences were confirmed by using SnapGene (GSL Biotech LLC).

### iPSC culture

In this study, 1383D6 (HPS1006 from RIKEN Cell Bank) was used as a healthy donor iPSC line since this line is transgene-free and widely used for various disease modeling studies. As juvenile nephronophthisis-specific hiPSC lines, HPS0447 (RIKEN Cell Bank) and HPS0450 (RIKEN Cell Bank) derived from patients were used. The culture hiPSCs were described in our previous studies (Shimizu et al., 2022; Song et al., 2022). Briefly, the iPSCs were cultured on 0.25 μg/cm^2^ iMatrix-511 silk (Matrixome) with StemFit AK02N medium (Ajinomoto) and passaged after 7 days of culture. The medium was changed every other day.

### Generation of NPHP1-overexpressing hiPSCs derived from patient’s hiPSC line

NPHP1-overexpressing hiPSCs derived from patient-derived hiPSC lines were generated by the PiggyBac transposase system. The patient-derived hiPSC lines, HPS0447 and HPS0450, were treated with TrypLE Express Enzyme (Thermo Fisher) and dissociated into single cells followed by resuspension with StemFit AK02N medium. The cell suspensions including 5 × 10^5^ cells were spun down in 1.5 mL tubes and were resuspended in 100 µL of Opti-MEM I Reduced Serum Medium (Gibco) including 7.5 µg of pPB-CAG-NPHP1-PGK-HygR (this paper, deposited to Addgene plasmid #209769) and 2.5 µg of pHL-EF1a-hcPBase-iP-A (RIKEN BRC DNA Bank) and subsequently transferred to the cuvette. The PiggyBac plasmid mixture in the cuvette was transduced into the hiPSC lines using the NEPA21 electroporator (NEPAGENE). The conditions of electroporation were as follows: [Parameter (unit), Poring Pulse/Transfer Pulse], [Voltage (V), 125/20], [Pulse width (ms), 5.0/50], [Pulse interval (ms), 50/50], [Number of pulses (time), 2/5], [Damping factor (%), 10/40], [Polarity, +/±]. After electroporation, the hiPSCs were transferred to culture on 0.25 μg/cm^2^ iMatrix-511 silk with StemFit AK02N medium including 10 µM Y-27632 (WAKO) followed by the medium change with only StemFit AK02N medium on next day. Transfected cells were selected by 200 μg/mL of hygromycin and maintained by 100 μg/mL of hygromycin after the selection.

### Generation of NPHP1-deficient hiPSCs derived from healthy donor

To obtain NPHP1-deficient hiPSCs derived from a healthy donor hiPSC line, the synthetic RNP complex of S.p. Cas9 and gRNA targeting exon1 of the *NPHP1* gene was introduced into 1383D6 line. The gRNA was designed with CRISPRdirect online software (https://crispr.dbcls.jp/) to minimize the off-target sequences and was synthesized by hybridizing equal amounts of 10 µM crRNA designed for including the *NPHP1* gene-specific site and 10 µM tracrRNA at 95°C for 5 min. The target sequence of crRNA was 5′-GGC​CCT​GCG​GCG​CCG​CAA​TC-3’. The RNP complex was generated by incubating 50 µL of synthesized 5 µM gRNA and 1 µL of 62 µM Alt-R S.p. HiFi Cas9 Nuclease V3 (Integrated DNA Technologies) at 25°C for 5 min. The generated RNP complex was diluted with 49 µL of Opti-MEM I Reduced Serum Medium. The healthy donor hiPSCs 1383D6, which were dissociated into the single cells using TrypLE Express Enzyme and subsequently prepared for 2.5×10^5^ cells in a 1.5 mL tube, were resuspended with the 100 µL of RNP solution and transfected by electroporation as described above. The transfected hiPSCs were cultured on 0.25 μg/cm^2^ iMatrix-511 silk with StemFit AK02N medium including 10 µM Y-27632 (WAKO), followed by the medium change with only StemFit AK02N medium on next day. The hiPSCs were cultured on a 6-well plate until semi-confluent.

### T7E1 assay

The insertions or deletions in exon1 of *NPHP1* were detected with the T7E1 assay. After sufficient proliferation, the hiPSCs were dissociated into single cells using TrypLE Express enzyme, and then 1.0 × 10^6^ cells were transferred to 1.5 mL tube. To obtain the PCR products around the genome editing site of *NPHP1*, the direct PCR of the hiPSCs was performed using Terra PCR Direct Polymerase Mix (TAKARA). The sequences of direct PCR primers are listed in Supplementary Table S2. The T7E1 assay was performed using Guide-it Mutation Detection Kit (TAKARA) following the manufacturer’s protocol. The DNA fragments were detected by MultiNA.

### Cloning and genotyping of hiPSCs

To clone the single cells with the indels of *NPHP1*, after the T7E1 assay, dissociated 1,000 cells were seeded in a 100 mm dish with StemFit AK02N medium supplemented with 10 µM Y-27632 and 0.25 μg/cm^2^ iMatrix-511 silk followed by the medium change on the next day. After several days of culture, the colonies derived from a single cell were picked and transferred to a 24-well plate. The cloned cells were cultured on a 24-well plate for around 1 week and transferred to a 6-well plate for proliferation culture. To examine the mutation of cloned hiPSCs, genome DNA was extracted using DNeasy Blood & Tissue Kit (QIAGEN). The PCR was performed using Tks Gflex DNA Polymerase (TAKARA) with the same primers used for direct PCR. The PCR products were purified using FastGene Gel/PCR extraction kit (Nippon Genetics) followed by Sanger sequencing at FASMAC. The sequences were analyzed by using SnapGene. The NPHP1-KO clonal lines #1 – #4 have been deposited in RIKEN cell bank as HPS5830, HPS5831, HPS5832, HPS5833, respectively.

### Quantitative RT-PCR

Total RNA extraction and cDNA synthesis were the same as above. Quantitative real-time PCR was performed with a QuantStudio 3 System (Applied Biosystems) using THUNDERBIRD Probe qPCR Mix (Toyobo) with TaqMan probes following the manufacturer’s instructions. The thermal cycling conditions were as follows: initial denaturation at 95°C for 20 min, 50 cycles of 2-step thermo-cycling (denaturation at 95°C for 1 s, annealing and extension at 60°C for 20 s), and then hold at 4°C. The gene expression levels were shown as ΔΔCt method normalized to corresponding GAPDH values. The TaqMan probes used in this study are Hs01019996_m1 for GAPDH and Hs02786624_g1 for NPHP1 (Thermo Fisher Scientific).

### Immunocytochemistry of hiPSCs

The hiPSCs were seeded on a 35 mm glass-base dish pre-coated with 0.5 μg/cm^2^ iMatrix-511 silk for 1 h. On day 5 after seeding, the hiPSCs were fixed with 4% paraformaldehyde for 20 min at 25°C and then permeabilized with 0.1% triton X-100 for 10 min at 25°C. After permeabilization, the cells were washed with DPBS (Nacalai tesque) and then incubated with primary antibody diluted in 0.1% donkey serum in DPBS overnight at 4°C. The cells were washed with DPBS for 5 times and then incubated with a secondary antibody diluted in 0.1% donkey serum in DPBS for 1 h at 25°C. After incubation, the cells were washed with DPBS for 5 times. The nuclei were stained with Fluoro-KEEPER Antifade Reagent with DAPI (Nacalai tesque). Images were taken with an all-in-one fluorescent microscope (BZ-X800; KEYENCE). The primary and secondary antibodies used in this study are listed in Supplementary Tables S3, S4.

### Western blot

Western blot was performed with a capillary automatic Western blot device (Simple Western, Wes; ProteinSimple) following the manufacturer’s instructions. 1.0×10^6^ cells were prepared with SDS-PAGE Sample Buffer Solution without 2-ME (2x) (Nacalai Tesque) supplemented with 100 mM Dithiothreitol solution (Nacalai Tesque) and were incubated for 5 min at 95°C. The following primary antibodies were used to blot the target protein: anti-GAPDH (1:500) and anti-NPHP1 (1:500). Detailed information on the primary antibodies is listed in Supplementary Table S3. The chemiluminescent signal was detected and quantitated automatically by a Simple Western system. The Compass software for Simple Western (Protein Simple) was used to process and analyze the data. The relative protein expression levels were normalized with each sample’s GAPDH protein expression level.

### Analysis of primary cilia

To analyze primary cilia, immunostaining of cilia-related protein ARL13B was performed for visualizing cilia. Fluorescent images of cilia were taken with an x100 lens of an all-in-one fluorescent microscope. The length of cilia and the ciliated cell ratio were measured by using Keyence BZ-X800 Analyzer (version 1.1.1.8).

### Generation of kidney organoids from hiPSCs

Kidney organoids were generated from hiPSCs referring to previous reports with minor modifications (Freedman et al., 2015). hiPSCs with semiconfluent state 7 days after passage were detached from the dish using TrypLE Express enzyme. After scraping and pipetting, 6.0×10^5^ cells were seeded onto a well of a 6-well plate pre-coated with 5% Geltrex (Gibco) in 1.5 mL DPBS and cultured overnight in 3 mL of StemFit AK02N medium with 10 µM Y-27632. On the next day, the medium was changed to 3 mL of StemFit Ak02N including 2.5% Geltrex. After 24 h, the medium was replaced with only 3 mL of StemFit AK02N. The next day (day 0), hiPSC spheroids were treated with 12 µM CHIR99021 in 5 mL of RPMI 1640 (Nacalai tesque) for 36 h. After exposure to the high concentration of CHIR99021, the cells were cultured with 3 mL of RPMI 1640 with 1X B27 supplement (Gibco) until day 18. The medium was changed every other day.

### Hematoxylin and eosin (HE) staining on the sections of kidney organoids

Kidney organoids were fixed with 4% paraformaldehyde for overnight at 4°C. Then, these organoids were embedded on CT-Pro20 (Genostaff Co., Ltd.) using G-Nox (Genostaff Co., Ltd.) as a less toxic organic solvent than xylene. The tissue blocks were sectioned at 10 µm and stained with HE. Sectioning and HE staining were performed at Genostaff inc.

### Immunocytochemistry of kidney organoids

Kidney organoids on day 18 were fixed with 4% paraformaldehyde for 15 min at 25°C and then permeabilized with 0.2% triton X-100 in DPBS (PBS-T) for 15 min at 25°C. After permeabilization, the organoids were incubated with 10% donkey serum in PBS-T (blocking buffer) for 1 h at 25°C. After blocking treatment, the organoids were incubated with primary antibodies diluted in blocking buffer overnight at 4°C. After washing by DPBS, the organoids were incubated with secondary antibodies diluted in blocking buffer overnight at 4°C. After wash, DPBS including one portion of Fluoro-KEEPER Antifade Reagent, Non-Hardening Type with DAPI was added to the well. Images were taken with an all-in-one fluorescent microscope. The primary and secondary antibodies used in this study are listed in Supplementary Tables S3, S4, respectively.

### Suspension culture of kidney organoids

On day 18, kidney organoids were detached from the well while maintaining nephron structures using a needle. A detached organoid was transferred to 2 mL of RPMI1640 with 1X B27 in a well of a low adherence 6-well plate (IWAKI) using a 200 µL large bore tip (WATSON). The organoids in 6-well plates were incubated at 37°C and 5% CO_2_ on the shaker at 90 rpm in an incubator for 2 weeks. The medium was changed every other day.

### RNA-seq

The total RNA of 12 samples was extracted from each whole dish with the FastGene RNA premium kit (Nippon Genetics). Strand-specific library preparation was performed. The prepared library was sequenced by a NovaSeq/HiSeq (Illumina). Sequencing was performed in a 2 × 150-bp PE configuration with a data output of about 6 Gb per sample. All the samples’ quality scores (Q30) were more than 94%. Library preparation and sequencing were performed in AZENTA. The sequencing data were analyzed with a CLC genomics workbench v20.0.2 (QIAGEN). GSEA was performed using the GSEA software v4.3.2 software. Gene ontology analysis was performed with extracted genes (FDR <0.25) using Metascape v3.5.20230501 (https://metascape.org/). Original data were deposited in NBDC database as Dataset ID: JGAS00683 (https://humandbs.biosciencedbc.jp/en/hum0302-v2#JGAS000382).

### Statistical analysis

The data are the mean ± the standard error (SE) in graphs generated using the Microsoft Excel software program for Windows (version 2308; Microsoft) and the R software program (version 4.2.2). For the comparison of two samples, *p*-values were determined by an unpaired two-tailed Student’s t-test, and for the comparison of more than two samples, *p*-values were determined by Tukey’s test using the R software program (version 4.2.2). For cystogenesis experiments, *p*-values were determined by Pearson’s chi-square test using the Microsoft Excel software program for Windows (version 2308; Microsoft). *p*-values less than 0.05 were considered statistically significant.

## Data Availability

The datasets presented in this study can be found in online repositories. The names of the repository/repositories and accession number(s) can be found below: https://humandbs.dbcls.jp/en/hum0302-v2#JGAS000382.
