# Supplementary material for: Patient-derived and gene-edited pluripotent stem cells lacking NPHP1 recapitulate juvenile nephronophthisis in abnormalities of primary cilia and renal cyst formation
Source: Front Cell Dev Biol. 2024 Jun 26;12:1370723. doi: 10.3389/fcell.2024.1370723 (PMC11233770; doi:10.3389/fcell.2024.1370723)
Supplement: Supplementary file 1 [file DataSheet1.PDF]

Supplementary Material

1 Supplementary Figures and Tables

1.1 Supplementary Figures

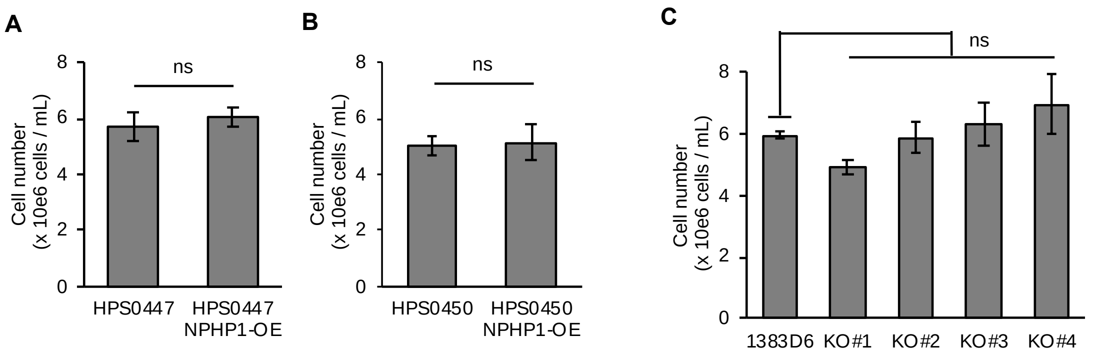

Supplementary Figure S1. The proliferation of NPHP1-deficient iPSCs used in this study, related to Fig 3. (A and B) Cell number of patients' hiPSCs HPS0447 and HPS0450, NPHP1-overexpressing hiPSCs HPS0447 NPHP1-OE and HPS0450 NPHP1-OE 7 days after previous passage. The experiments were repeated three times (n = 3). Data are shown as the mean ± SEM (n = 3). P-values were determined by an unpaired two-tailed Student's t-test. (C) Cell number of healthy donor hiPSCs 1383D6, NPHP1-deficient hiPSCs KO#1, KO#2, KO#3, KO#4 7 days after previous passage. The experiments were repeated three times (n = 3). Data are shown as the mean ± SEM (n=3). P-values were determined by Tukey's test.

A 1383D6 line

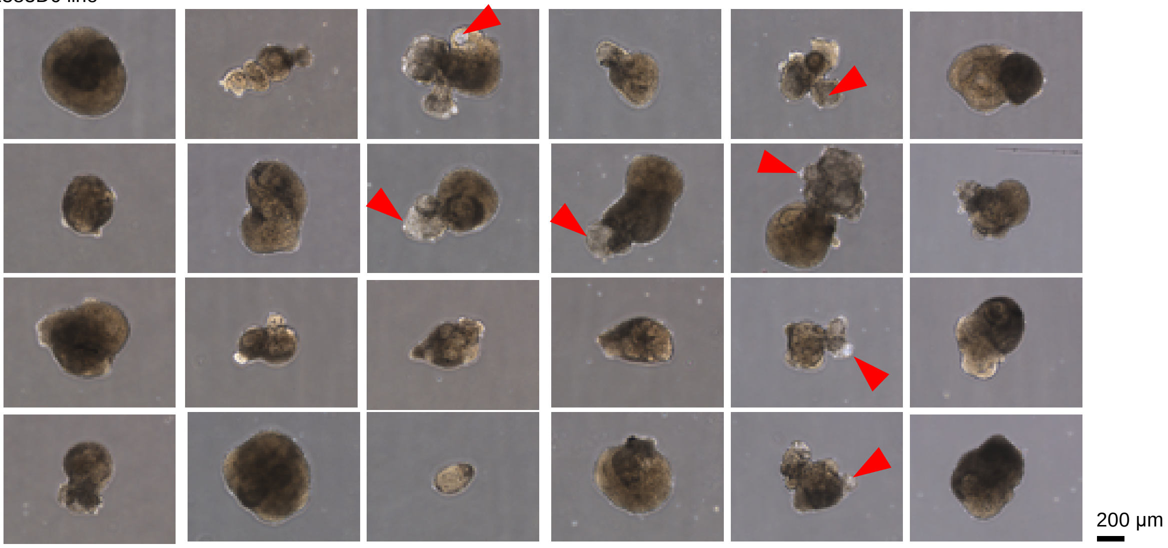

B NPHP1-KO line

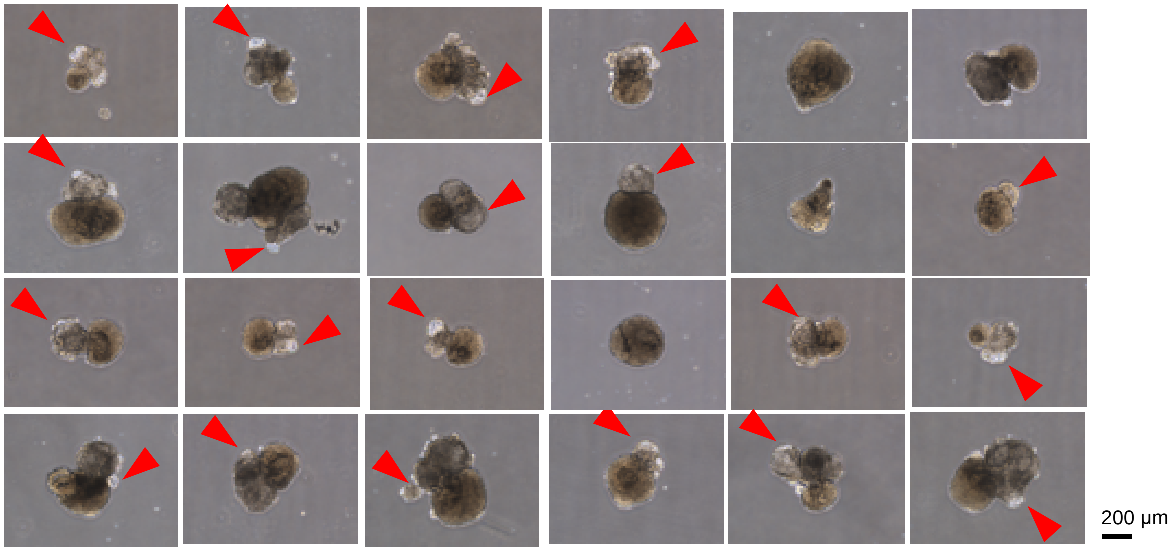

C HPS0450 line

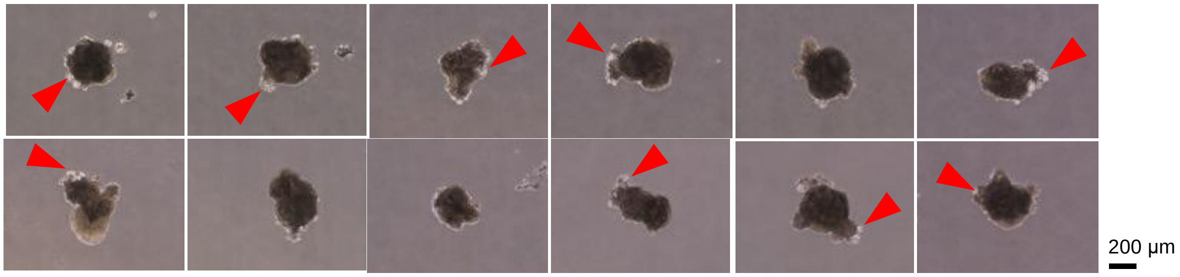

D HPS0450-OE line

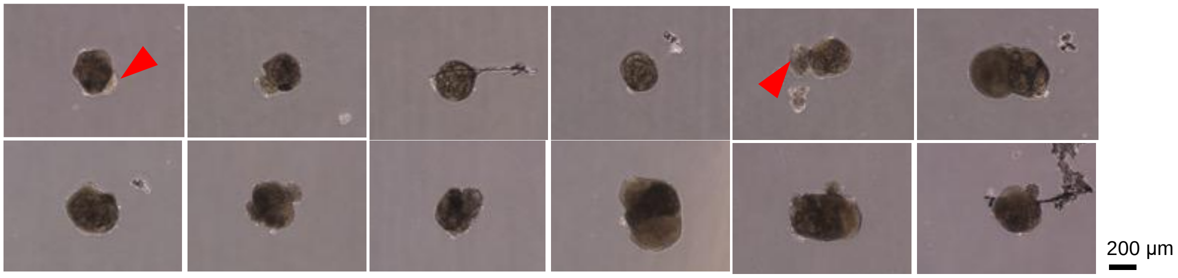

Supplementary Figure S2. Kidney organoids derived NPHP1-deficient hiPSCs recapitulate the renal cyst formation in suspension culture with rotation.

(A-D) Phase contrast images of kidney organoids in suspension culture from 1383D6 (A), NPHP1-KO#2 (B), HPS0450 (C), and HPS0450 NPHP1-OE (D) lines. Red arrowheads indicate renal cysts. Scale bars = 200 μm.

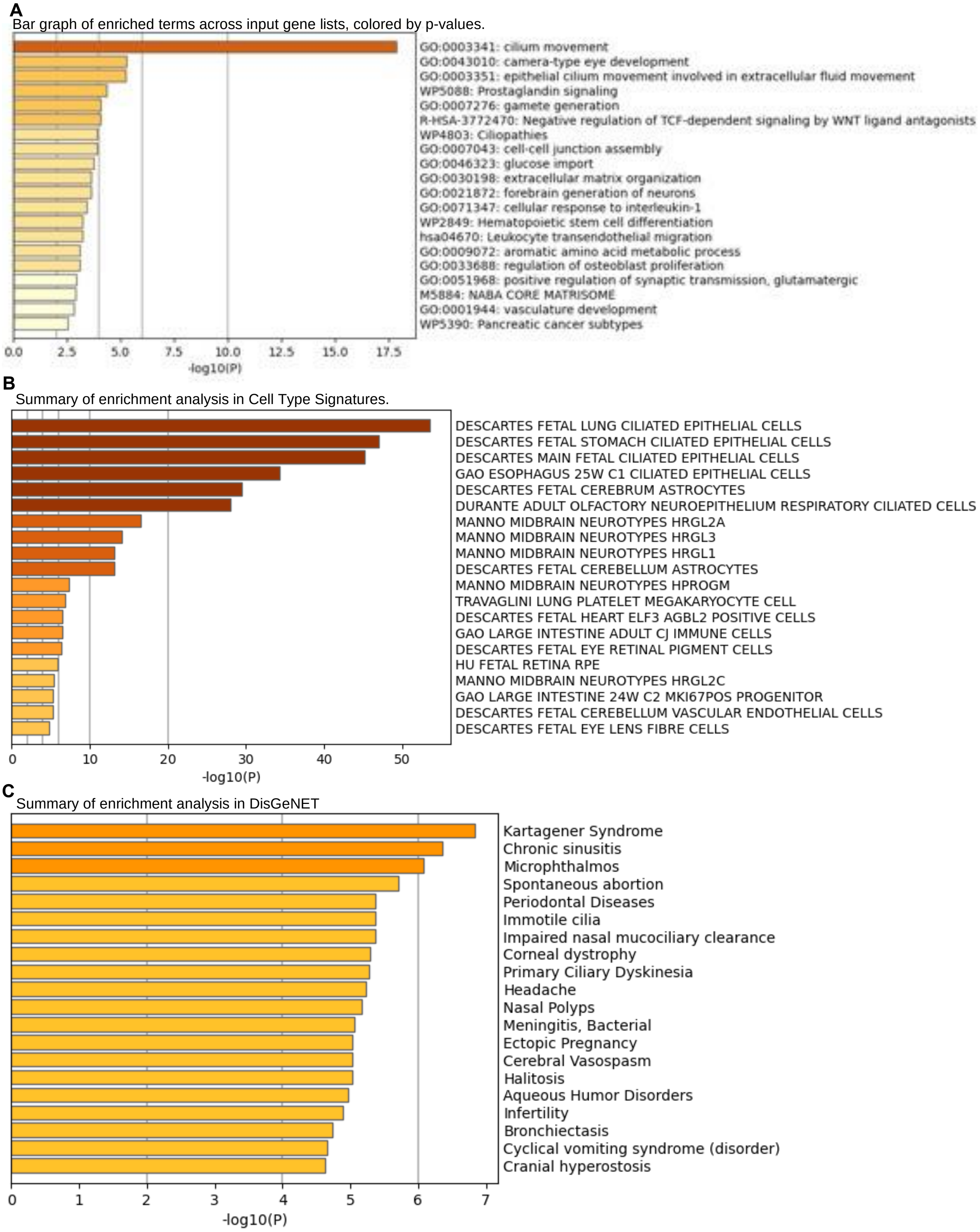
A
Bar graph of enriched terms across input gene lists, colored by p-values.
GO:0003341: cilium movement
GO:0043010: camera-type eye development
GO:0003351: epithelial cilium movement involved in extracellular fluid movement
WP5088: Prostaglandin signaling
GO:0007276: gamete generation
R-HSA-3772470: Negative regulation of TCF-dependent signaling by WNT ligand antagonists
WP4803: Ciliopathies
GO:0007043: cell-cell junction assembly
GO:0046323: glucose import
GO:0030198: extracellular matrix organization
GO:0021872: forebrain generation of neurons
GO:0071347: cellular response to interleukin-1
WP2849: Hematopoietic stem cell differentiation
hsa04670: Leukocyte transendothelial migration
GO:0009072: aromatic amino acid metabolic process
GO:0033688: regulation of osteoblast proliferation
GO:0051968: positive regulation of synaptic transmission, glutamatergic
M5884: NABA CORE MATRISOME
GO:0001944: vasculature development
WP5390: Pancreatic cancer subtypes
0.0 2.5 5.0 7.5 10.0 12.5 15.0 17.5
-log10(P)
B
Summary of enrichment analysis in Cell Type Signatures.
DESCARTES FETAL LUNG CILIATED EPITHELIAL CELLS
DESCARTES FETAL STOMACH CILIATED EPITHELIAL CELLS
DESCARTES MAIN FETAL CILIATED EPITHELIAL CELLS
GAO ESOPHAGUS 25W C1 CILIATED EPITHELIAL CELLS
DESCARTES FETAL CEREBRUM ASTROCYTES
DURANTE ADULT OLFACTORY NEUROEPITHELIUM RESPIRATORY CILIATED CELLS
MANNO MIDBRAIN NEUROTYPES HRGL2A
MANNO MIDBRAIN NEUROTYPES HRGL3
MANNO MIDBRAIN NEUROTYPES HRGL1
DESCARTES FETAL CEREBELLUM ASTROCYTES
MANNO MIDBRAIN NEUROTYPES HPROGM
TRAVAGLINI LUNG PLATELET MEGAKARYOCYTE CELL
DESCARTES FETAL HEART ELF3 AGBL2 POSITIVE CELLS
GAO LARGE INTESTINE ADULT CJ IMMUNE CELLS
DESCARTES FETAL EYE RETINAL PIGMENT CELLS
HU FETAL RETINA RPE
MANNO MIDBRAIN NEUROTYPES HRGL2C
GAO LARGE INTESTINE 24W C2 MKI67POS PROGENITOR
DESCARTES FETAL CEREBELLUM VASCULAR ENDOTHELIAL CELLS
DESCARTES FETAL EYE LENS FIBRE CELLS
0 10 20 30 40 50
-log10(P)
C
Summary of enrichment analysis in DisGeNET
Kartagener Syndrome
Chronic sinusitis
Microphthalmos
Spontaneous abortion
Periodontal Diseases
Immotile cilia
Impaired nasal mucociliary clearance
Corneal dystrophy
Primary Ciliary Dyskinesia
Headache
Nasal Polyps
Meningitis, Bacterial
Ectopic Pregnancy
Cerebral Vasospasm
Halitosis
Aqueous Humor Disorders
Infertility
Bronchiectasis
Cyclical vomiting syndrome (disorder)
Cranial hyperostosis
0 1 2 3 4 5 6 7
-log10(P)

Supplementary Figure S3. Transcriptome analysis of kidney organoids reveals that loss of NPHP1 causes primary cilia abnormalities in epithelial cells.

(A) A bar chart of candidate pathway and process of 211 down-regulated genes in the disease group selected by adjusted p values < 0.25. **(B)** A bar chart showing cell type signatures down-regulated genes. **(C)** A bar chart of quality control and association showing candidate disease types using the DisGeNET program for down-regulated genes.

1.2 Supplementary Tables

Table S1. The list of terms significantly down-regulated or up-regulated in GSEA.

NAME	Size	NES	FDR q-value	RANK AT MAX	LEADING EDGE
WP_CYTOPLASMIC_RIBOSOMAL_PROTEINS	88	-1.7979282	0.04415653	9636	tags=78%, list=24%, signal=103%
WP_CILIOPATHIES	182	-1.8026865	0.05009674	7814	tags=59%, list=20%, signal=74%
HOLLERN_EMT_BREAST_TUMOR_DN	121	-1.759004	0.09394621	7688	tags=67%, list=19%, signal=83%
SOTIRIOU_BREAST_CANCER_GRADE_1_VS_3_UP	153	2.0572	0	8036	tags=72%, list=20%, signal=90%
REACTOME_CHOLESTEROL_BIOSYNTHESIS	27	1.8790247	0.04821928	3957	tags=78%, list=10%, signal=86%
MANALO_HYPOXIA_DN	290	1.8260018	0.05521547	8367	tags=61%, list=21%, signal=77%
HORTON_SREBF_TARGETS	23	1.826827	0.06112794	4817	tags=83%, list=12%, signal=94%
WHITEFORD_PEDIATRIC_CANCER_MARKERS	115	1.8316594	0.06412023	8782	tags=73%, list=22%, signal=94%
WINNEPENNINCKX_MELANOMA_METASTASIS_UP	161	1.7979089	0.06805075	9003	tags=66%, list=23%, signal=86%
KOBAYASHI_EGFR_SIGNALING_24HR_DN	253	1.8083048	0.06814668	8595	tags=69%, list=22%, signal=87%
ZHOU_CELL_CYCLE_GENES_IN_IR_RESPONSE_6HR	83	1.8470755	0.06905995	7987	tags=70%, list=20%, signal=87%
BENPORATH_PROLIFERATION	146	1.7915344	0.07096669	9197	tags=70%, list=23%, signal=91%
SCHMIDT_POR_TARGETS_IN_LIMB_BUD_UP	27	1.8799095	0.07127023	4817	tags=89%, list=12%, signal=101%
REACTOME_SYNTHESIS_OF_DNA	120	1.8005636	0.07146505	8386	tags=66%, list=21%, signal=83%
WP_CHOLESTEROL_BIOSYNTHESIS_PATHWAY	15	1.8357848	0.07167462	3260	tags=100%, list=8%, signal=109%
KAUFFMANN_MELANOMA_RELAPSE_UP	61	1.7632984	0.08535354	8918	tags=69%, list=23%, signal=89%
MORI_LARGE_PRE_BII_LYMPHOCYTE_UP	88	1.7645096	0.08900283	9332	tags=74%, list=24%, signal=96%
BLANCO_MELO_BRONCHIAL_EPITHELIAL_CELLS_INFLUENZA_A_DEL_NS1_INFECTION_DN	185	1.7683609	0.08927592	8036	tags=59%, list=20%, signal=74%
REACTOME_MITOTIC_G1_PHASE_AND_G1_S_TRANSITION	148	1.7694511	0.09427187	9158	tags=73%, list=23%, signal=95%
REACTOME_APC_C_CDH1_MEDIATED_DEGRADATION_OF_CDC20_AND_OTHER_APC_C_CDH1_TARGETED_PROTEINS_IN_LATE_MITOSIS_EARLY_G1	73	1.7531075	0.09481973	9298	tags=71%, list=23%, signal=93%
ROSTY_CERVICAL_CANCER_PROLIFERATION_CLUSTER	139	1.7358589	0.09491032	9317	tags=73%, list=24%, signal=95%
WP_CHOLESTEROL_SYNTHESIS_DISORDERS	18	1.7320524	0.09551133	3957	tags=89%, list=10%, signal=99%
GRAHAM_NORMAL_QUIESCENT_VS_NORMAL_DIVIDING_DN	89	1.7377844	0.09657473	8290	tags=74%, list=21%, signal=94%
REACTOME_DNA_REPLICATION	165	1.7396291	0.09860841	7741	tags=59%, list=20%, signal=73%

Table S2. List of primers used in this study

**Name**	**Forward/Reverse primer (5′−3′)**
In-Fusion primer for NPHP1 cDNA fragment	F: ACGGTATCGATAAGCATGCTGGCGAGACGACAG<br>R: CACCACTTTGTACAATCACACTGCATTCTTTCTCATTTCACC
In-Fusion primer for linearized vector	F: TTGTACAAAGTGGTGATAGCTTGGC<br>R: GCTTATCGATACCGTCGAGGAATTC
pPB-CAG-NPHP1-PGK-HygR primer 1	GCTGAGCAGTAAAATCTCCAACAGCG
pPB-CAG-NPHP1-PGK-HygR primer 2	GGGCCTTGCTGTGACAATAAGCAGA
pPB-CAG-NPHP1-PGK-HygR primer 3	ATGTAGAGGCGGTGGATGAAACAGC
pPB-CAG-NPHP1-PGK-HygR primer 4	GCCTTCACAACTGGCCTTCAGAGAT
pPB-CAG-NPHP1-PGK-HygR primer 5	GAGTTAAGCTGTGGCTGGGTGTTTCT
pPB-CAG-NPHP1-PGK-HygR primer 6	CAGGAGTTCGTGGGCTGGAAAAGAA
Direct PCR primer for NPHP1 cDNA	F: CAGGTATCCCAGCAAGAAGGTGGTG<br>R: AGGCTCAGAGTTGGGTTGGAAATGG

Table S3. The list of primary antibodies used in this study.

**Primary Abs**	**Host species**	**Dilutions**	**SOURCE**	**IDENTIFIER**
NPHP1	Rabbit	1:500 (WB) / 1:2000 (ICC)	Abcam	Cat#ab211422
GAPDH	Mouse	1:500	R&D systems	Cat#MAB5718
Acetylated-tubulin	Mouse	1:2000	Sigma-Aldrich	Cat#T6793
ARL13B	Rabbit	1:800	Proteintech	Cat#17711-1-AP
NPHS1	Sheep	1:300	R&D systems	Cat#AF4269
LRP2	Rabbit	1:200	Abcam	Cat#ab76969
ECAD	Mouse	1:300	BD Biosciences	Cat#610181

Table S4. The list of secondary antibodies used in this study.

Secondary Abs	Dilution	SOURCE	IDENTIFIER
Anti-Mouse Secondary HRP Antibody	Not diluted	ProteinSimple	Cat#042-205
Anti-Rabbit Secondary HRP Antibody	Not diluted	ProteinSimple	Cat#042-206
DyLight™ 649 Donkey anti-rabbit IgG (minimal x-reactivity) Antibody	1:500	Biolegend	Cat#406406
Donkey anti-Mouse IgG (H+L) Highly Cross-Adsorbed Secondary Antibody, Alexa Fluor™ 488	1:500	ThermoFisher Scientific	Cat#A-21202
Donkey anti-Sheep IgG (H+L) Cross-Adsorbed Secondary Antibody, Alexa Fluor™ 647	1:500	ThermoFisher Scientific	Cat#A-21448
DyLight™ 488 Donkey anti-rabbit IgG (minimal x-reactivity) Antibody	1:500	Biolegend	Cat#406404
Donkey anti-Mouse IgG (H+L) Highly Cross-Adsorbed Secondary Antibody, Alexa Fluor™ 555	1:500	ThermoFisher Scientific	Cat#A-31570
